# Effects of Daily Peat Smoke Exposure on Present and Next Generations

**DOI:** 10.3390/toxics10120750

**Published:** 2022-12-01

**Authors:** Vera A. Vokina, Larisa M. Sosedova, Mikhail A. Novikov, Evgeniy A. Titov, Elizaveta S. Andreeva, Viktor S. Rukavishnikov

**Affiliations:** FSBSI East-Siberian Institute of Medical and Ecological Research, 665826 Angarsk, Russia

**Keywords:** peat smoke, rats, offspring, behavior

## Abstract

This study aimed to follow the neurotoxic effect of peat smoke on adult outbred rats and its influence on central nervous system (CNS) parameters in first-generation offspring. Under experimental conditions, exposure to peat smoke was carried out on adult male Wistar rats for 24 h. After the end of the exposure, an open field test (OFT), electroencephalography (EEG), and histological analysis of the testes and brains of smoke-exposed males were performed, after which they were mated with intact females to obtain F1 offspring. Stillbirth, neonatal mortality, and body weight at 4, 7, 14, and 21 postnatal days, as well as behavior in the OFT and EEG parameters during puberty (3 months), were assessed. The results of the examination of F0 males showed a significant increase in motor activity and anxiety in the open field test and a violation of EEG parameters. Histopathologically, peat smoke caused a sharp increase in shadow cells (homogeneous cells with pale-stained cytoplasm, in which the cell and nuclear membranes are not visualized) and degeneratively altered neurons in the brain; we found no changes in the testicles. Peat smoke exposure during preconception did not affect neonatal mortality and weight gain in F1 offspring. Adult females born to peat-smoke-exposed males showed an increase in locomotor activity, and the behavior of adult F1 males did not differ from the control. In F1 males, a statistically significant increase in slow-wave activity indices in the delta band was observed.

## 1. Introduction

Large peat fires, which have become regular events in the modern world, are expanding to a global scale [1,2,3]. A distinctive feature of peat fires is their duration from a week to 5 or more years, their occurrence in any period of the year, and the extreme difficulty of extinguishing them. At the same time, toxic smoke from burning peat with a high content of fine particles and polyaromatic hydrocarbons, including methane, aldehydes, organic acids, phenols, and other organic substances, spreads over long distances and crosses geographical boundaries, affecting areas remote from the original source [4].

Exposure to smoke from peat fires poses a serious health hazard to both the public and firefighters [5]. Particulate air pollution (PM_2.5_) is often used as a criterion for the intensity of exposure to wildfire smoke [6]. The ability of PM_2.5_ to reach deep into the lungs and cross the air/blood–brain barrier makes it extremely dangerous to human health, leading not only to the development or aggravation of respiratory diseases but also to extrapulmonary health effects, including effects on the heart, brain, and reproductive organs [7,8,9,10]. Despite the fact that the activity of forest fires is increasing and cases of smoke generation are becoming more frequent and prolonged, their impact on the state of the central nervous and reproductive systems, as well as the mechanisms of the development of long-term effects in offspring, remains poorly understood.

This investigation was devoted to the study of the effect of peat smoke on the state of the central nervous system and the reproductive potential of male white rats, as well as the survival, postnatal growth, and CNS parameters of their F1 offspring.

## 2. Materials and Methods

### 2.1. Animals and Experimental Design

The study was conducted in accordance with the rules for the humane treatment of animals following the requirements of the International Guidelines for Biomedical Research Using Animals (WHO, Geneva, Switzerland, 1985), the UK Animal Law (Scientific Procedures) (UK, 1986), and the guidelines of the National Institutes of Health on the Care and Use of Laboratory Animals (NIH Publication No. 8023, revised 1978). All animal experiments were approved by the ethics committee of the East Siberian Institute for Medical and Ecological Research (identification code: E41/20; approval date: 5 October 2020, changes/approvals every 6 months).

The experimental animals were obtained from the vivarium of the East Siberian Institute of Medical and Ecological Research (ESIMER) and were kept on a standard diet (BioPro Russia, ad libitum water). Animals were kept under a 12/12 h light/dark cycle on a ventilated shelf and under controlled temperature and humidity conditions (22–25 °C and 55–60% humidity).

Wistar male rats (*n* = 30) were exposed to inhalation smoke continuously for 24 h. Rats of the control group (*n* = 30) were supplied with clean air into the chamber. One day after the end of the exposure, the animals were examined, which included an assessment of the CNS (open field test, electroencephalography, and histological examination of the tissue of the cerebral cortex) and the reproductive system (histological examination of the testis).

To obtain offspring of the first generation (F1), immediately after exposure, exposed males (*n* = 10) were mated with intact females in a ratio of 1:2. Two days before the expected date of birth, the females were seated in separate cages. The rat pups of all groups were weaned from their mothers on the 30th day of life. Stillbirths, neonatal mortality, and pup weights were assessed at 4, 7, 14, and 21 postnatal days (P4, P7, P14, and P21). The percentage of stillbirths was calculated as follows: Percentage of stillbirths = (Number of stillbirths/Total number of births in the group) × 100%. The death of newborn pups (except for stillborn ones) within 7 days after calving was taken into account as neonatal mortality. The percentage of neonatal mortality was calculated as follows: Percentage of neonatal mortality = (Number of neonatal deaths/Total number of births in the group) × 100%.

In adult F1 rats (P90), behavior (OFT) and EEG scores were evaluated. In total, 225 rat pups were examined in the neonatal period along with 80 adult F1 rats.

### 2.2. Exposure Study

Methodological approaches to modeling the impact of wildfire smoke with the identification of individual components of smoke and characterization of the air environment in the exposure chamber were described in detail by us earlier [11]. Briefly, experimental modeling of peat smoke intoxication was carried out in 200 L exposure chambers, in which rats were placed and peat smoke was supplied. Smoke was generated in a smoke generator in which peat smoldered.

Peat samples were taken from drained peat bogs in Angarsk (Irkutsk region, Russia). Smoke was diluted with clean air to achieve target concentrations of PM_2.5_ and CO (actual concentrations are shown in Table 1). Target concentrations of PM_2.5_ and CO were in the range of actual instrumental measurements that firefighters face when fighting forest fires [12,13]. Air samples were analyzed once per hour during exposure. The mass concentration of PM_2.5_ was measured with the piezo-balanced dust sensor Kanomax 3521 (Kanomax, St Paul, MN, USA). The carbon monoxide (CO) concentration was analyzed on a Chromos GC-1000 gas chromatograph (Chromos Engineering, Moscow, Russia), and NO_2_ and SO_2_ concentrations were measured using a PE-5300VI spectrophotometer (Ekroskhim, St. Petersburg, Russia).

### 2.3. Open Field Test

Testing was carried out in a round arena with a diameter of 40 cm, with a translucent floor, walls 25 cm high, and inverted lighting using the three-dimensional animal tracking system EthoStudio (Russia) [14]. Rats (peat-smoke-exposed group *n* = 20; control group *n* = 20) were placed near the wall and for 3 min were automatically assessed for the total distance traveled (m), the time (%) of the animal being in the central part of the arena (the time in the center was expressed as a percentage of the total observation time), and area of the explored arena (calculated as the fraction of the arena (%) visited by the rat), while the number of freezing events was manually recorded.

### 2.4. EEG Measurements

For local anesthesia and immobilization during electrode placement and EEG recording (peat-smoke-exposed group *n* = 10; control group *n* = 10), rats were injected subcutaneously with 0.1% solution of medetomidine hydrochloride (Apicenna, Balashikha, Russia) at a dose of 0.01 mg/kg. The EEG was recorded in a darkened quiet room using the portable electroencephalograph “Neuron-Spectrum-1/V” (Neurosoft, Ivanovo, Russia). Thin needle electrodes were used: (1) two recording electrodes were inserted subcutaneously into the parietal part of the head, into the left and right parts of the brain; (2) the reference electrode was inserted subcutaneously into the region of the nasal bone; and (3) the ground electrode was attached to the tail. The EEG was recorded in the background test mode for 60 s. The boundaries of the filters of high and low frequencies were 0.5 and 35 Hz, respectively; and the sampling rate was 200 s^−1^. Automatic removal of artifacts was used to exclude epochs with remaining artifacts. The clean epochs were subjected to a fast Fourier transform (FFT) to obtain the absolute spectral power in delta (0.5–4.0 Hz), theta (4.0–8.0 Hz), alpha (8.0–13.0 Hz), beta 1 (13.0–22.0 Hz), and beta 2 (22.0–32.0 Hz) frequency bands. The duration of the analysis epochs was 10 s. Spectral analysis was performed using the Neuron-Spectrum.NET program (Neurosoft, Ivanovo, Russia). The average amplitude of the spectrum in a certain frequency range (alpha, beta, theta, and delta rhythms) and the rhythm index (the percentage of time during which EEG activity is present in the EEG sample) were calculated separately in each specific range based on the power spectrum obtained based on the fast Fourier transform algorithm.

### 2.5. Methods of Histological Research

F0 males were euthanized by decapitation 24 h after exposure (peat-smoke-exposed group *n* = 10; control group *n* = 10). Testes (left) and brains were removed and fixed in neutral buffered formalin solution (10%), dehydrated with ascending concentrations of ethanol (70, 80, 90, 95, and 100%), and placed in a homogenized paraffin medium, HistoMix (BioVitrum, Petersburg, Russia), for histological studies. Then, layer-by-layer serial tissue sections 5 µm thick were prepared [15]. Section preparations were stained with hematoxylin–eosin to perform survey microscopy. Then, the sections were visualized using an Olympus BX 51 light-optical research microscope (OlympusCo, Tokyo, Japan) at a magnification of X100. The spermatogenesis index (the sum of all spermatogenesis cell stages calculated in 100 sections) and the number of normal spermatogonia were determined from testis histological slides. The functional state of the tissue of the sensorimotor cortex was judged by the change in the total number of neurons, the number of degenerative neurons and glial cells, and the number of shadow cells. Dark-stained deformed neurons without a clearly distinguishable nucleus and cytoplasm were considered degenerative. Animals were euthanized by decapitation.

### 2.6. Statistical Analysis

Statistical analysis of the results of the study was carried out using the Statistica 6.1 software package. The Shapiro–Wilk W test was used to determine the type of feature distribution. We used the Mann–Whitney U test to compare groups. Null hypotheses about the absence of differences between groups were rejected at a significance level of *p* ≤ 0.05.

## 3. Results

### 3.1. Exposure Characteristics

The content of solid PM_2.5_ particles in the exposure chamber ranged from 340 to 1420 µg/m^3^, and the average concentration of PM_2.5_ was 920 ± 80 µg/m^3^ (Table 1). The mean CO concentration was 40.8 ± 1.9 mg/m^3^. The concentration of nitrogen dioxide in the exposure chamber was 37 ± 2 µg/m^3^, and the content of sulfur dioxide did not exceed 5 µg/m^3^ (Table 1). 

### 3.2. Result of Behavioral Test

Exposure to peat smoke for 24 h led to a change in the behavior of rats in an open field.

**Figure 1:**
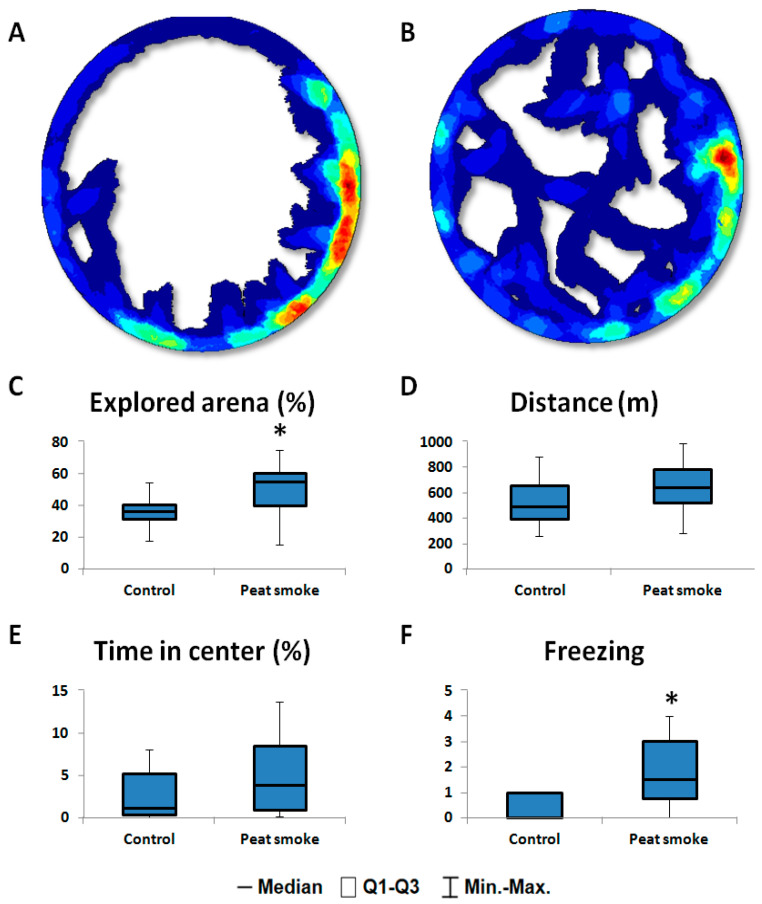
Effects of peat smoke exposure in F0 rats in an OFT. Representative images of animal activity during testing (red—highest activity; blue—lowest activity): (**A**) Control group; (**B**) rats exposed to peat smoke; (**C**) Area of the explored arena (%); (**D**) Total distance; (**E**) Time elapsed at the center; (**F**) Number of freezing episodes. Data are presented as median and IQR. * *p* < 0.05.

In the open field, peat-smoke-exposed rats showed a statistically significant increase in the studied area of the arena compared to the control group (*p* = 0.011; Figure 1), and in addition, the distance traveled by the exposed animals during testing tended to increase (*p* = 0.092; Figure 1). A significant increase in the level of anxiety in smoke-exposed rats was revealed, which manifested itself in an increase in the number of episodes of freezing (*p* = 0.048; Figure 1) compared with the control group. Along with the increase in freezing acts, the observed increase in motor activity in experimental animals should be considered an indicator of a high level of stress and impaired inhibitory processes.

### 3.3. EEG Analysis

**Figure 2:**
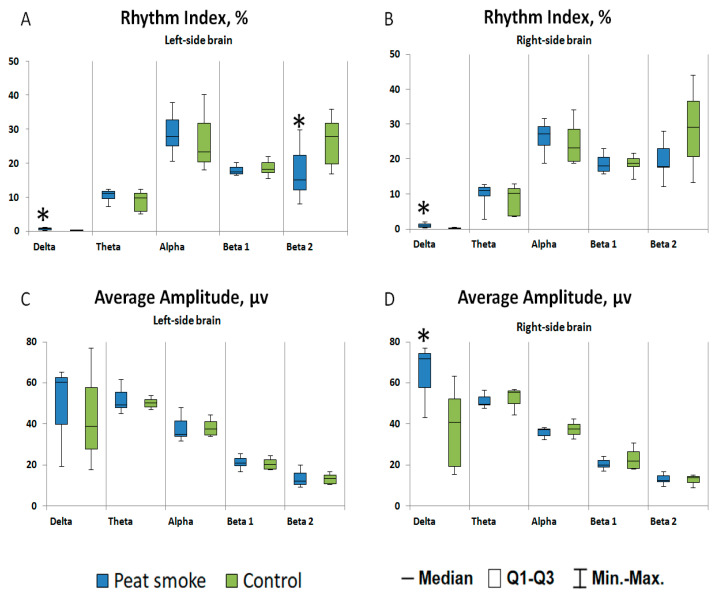
EEG results: rhythm indices of the left (**A**) and right (**B**) hemispheres; average amplitude of rhythms of the left (**C**) and right hemispheres (**D**). * *p* < 0.05.

The EEG results of rats showed that after acute daily exposure to peat smoke, there was a statistically significant increase in the index in both hemispheres and in the average amplitude of the δ-rhythm in the right hemisphere, (*p* = 0.006 and *p* = 0.006, respectively, Figure 2), and there was a decrease in the beta-2 rhythm index in the left hemisphere compared with the control group (*p* = 0.026, Figure 2). A trend towards a decrease in the average EEG amplitude of the θ-rhythm (*p* = 0.056, Figure 2) was revealed in the right hemisphere.

### 3.4. Histopathological Examination

**Figure 3:**
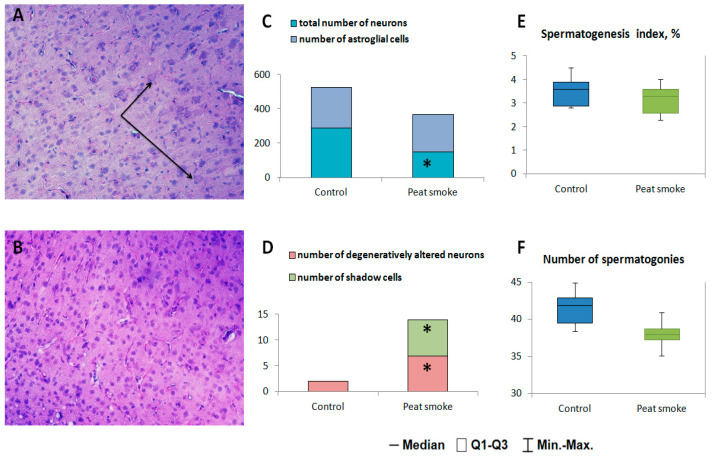
Results of histological investigation of the brain and testes of male F0 rats. Microphoto of brain tissue of control rats (**A**) and peat smoke (**B**): ↑—shadow cells. Hematoxylin–eosin stain. Mag. X100; (**C**) Number of normal neurons and astroglial cells; (**D**) Number of degeneratively altered neurons and shadow cells; (**E**) Index of spermatogenesis; (**F**) Number of spermatogonia. Data are presented as median and IQR. * *p* < 0.05.

A morphometric study showed a decrease in the total number of neurons of the cerebral cortex per unit area when exposed to peat smoke (*p* = 0.007, Figure 3C). The number of astroglial cells did not have a statistically significant difference from the control group. There was also an increase in the number of degeneratively altered neurons compared to control values (*p* = 0.04, Figure 3D). At the same time, a sharp increase in shadow cells was noted in the tissue of the sensorimotor cortex (*p* = 0.004, Figure 3A,B). The cytoplasm of shadow cells was pale-colored, homogeneous, and without clearly distinguishable contours of the nucleus and nucleoli. Some of the shadow cells were swollen and significantly increased in size.

Histological analysis of the testes of male rats exposed to peat smoke did not reveal significant changes in the spermatogenesis index and the number of spermatogonia when compared with the control group (Figure 3E,F).

### 3.5. Results of Examination of F1 Offspring from Peat-Smoke-Exposed Rats

An analysis of postnatal mortality showed that among F1 rat pups born from peat-smoke-exposed male rats, cases of stillbirth and death were 4.6% and 3.2%, respectively. In the control group, these figures were 0% and 2.8%, respectively.

**Figure 4:**
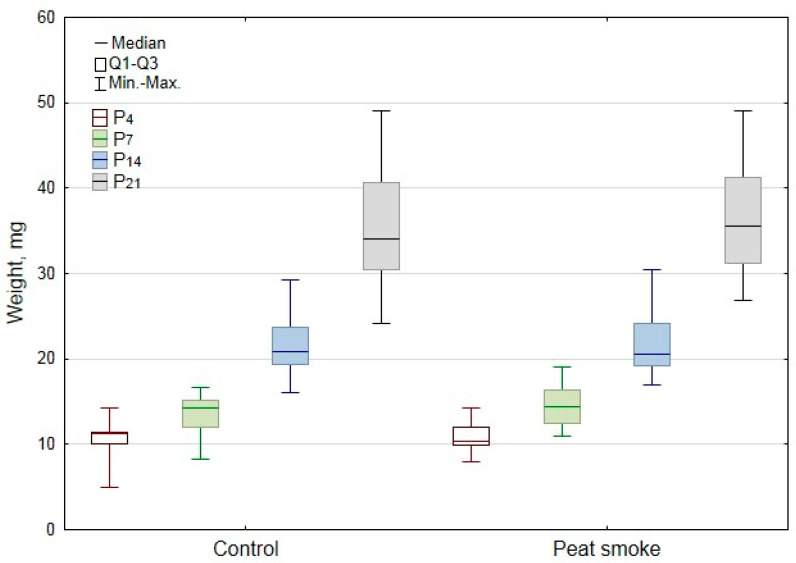
Body weight of newborn offspring on postnatal days P4, P7, P14, and P21.

The body weight of F1 newborn rat pups born from peat-smoke-exposed male rats on postnatal days P4, P7, P14, and P21 had no statistically significant differences when compared with the control group (Figure 4).

**Figure 5:**
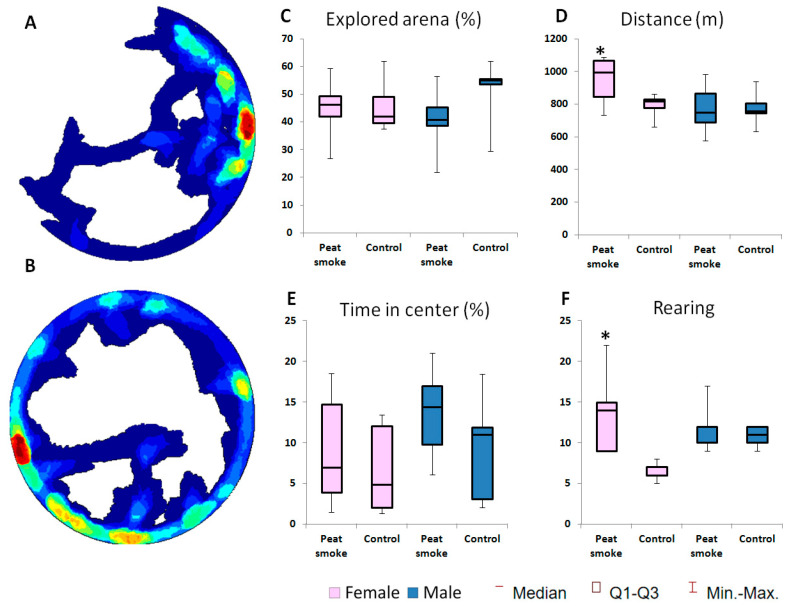
Effects of paternal peat smoke exposure on F1 rats in an OFT. Representative images of animal activity during testing (red—the highest activity; blue—the lowest): (**A**)—Control group; (**B**)—Peat-smoke-exposed rats; (**C**) Area of the explored arena (%); (**D**) Total distance covered in the open field test; (**E**) Time elapsed at the center; (**F**) Number of rearing. * *p* < 0.05.

Adult F1 rats were tested in the open field test, and electroencephalographic examination was carried out. In F1 offspring obtained from males exposed to natural fire smoke for 1 day, pronounced gender differences in the structure of behavior in an open field were revealed. Females showed a pronounced activation of locomotor activity, as evidenced by a statistically significant increase in both horizontal (distance) and vertical activity (rearing) compared with control indicators (*p* = 0.033 and *p* = 0.003, respectively, Figure 5). The behavior of F1 offspring males had no statistically significant differences when compared with the control group.

**Figure 6:**
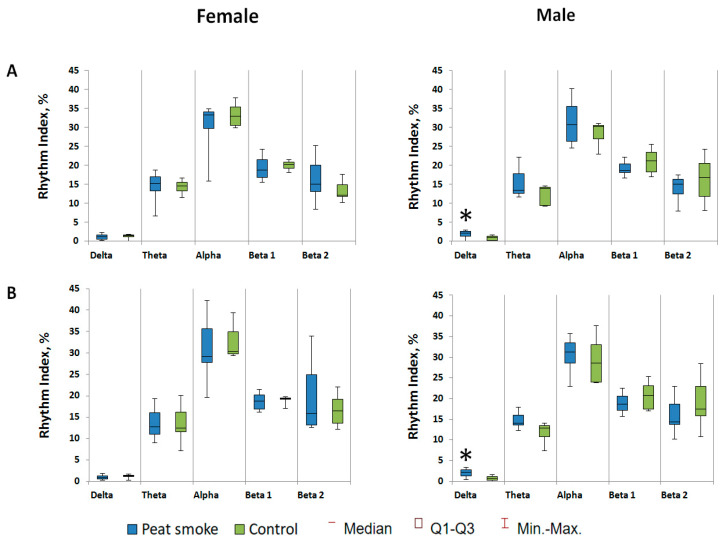
EEG parameters of F1 offspring: rhythm indices of the left (**A**) and right (**B**) hemispheres. * *p* < 0.05.

The results of the EEG examination showed that the male offspring had a statistically significant increase in slow-wave activity indices in the delta range in the left and right hemispheres (*p* = 0.030 and *p* = 0.049, respectively; Figure 6).

## 4. Discussion

Understanding the impact of wildfires on public health is an urgent task for the modern scientific community. Along with forested areas, large peatlands are highly susceptible to fire, and although less common, peat fires have serious impacts on ecosystems and human health. However, the health effects associated with the smoke of peat fires are much less studied than those of forest fires. Wildfire-specific PM_2.5_ is known to be 10 times more harmful than non-smoke PM_2.5_, and wildfires can often cause PM_2.5_ concentrations to spike [16,17], often exceeding safe limits (35 µg/m^3^) and reaching levels defined as hazardous (>250 µg/m^3^) by the Air Quality Index (AQI, USA). Burning peat releases more PM_2.5_ than any other type of biomass fuel source [18]. In addition, peat combustion in stoves for heating and cooking is a major source of peat-related indoor air pollution and a leading health risk in Asia, Africa, and Central/South America [19,20]. The changing world scenario in the context of global climate change, when PM_2.5_ is predicted to increase from wildfires compared to emissions from other sources, determines the need to study the toxic effects of wildfire smoke on various human organs and systems. Despite the available epidemiological data proving the impact of peat smoke on the development of adverse health effects, many mechanisms for the formation of these effects remain unexplored.

In the present study, rats were exposed to peat smoke once for 24 h at concentrations of PM_2.5_ and CO similar to those encountered by firefighters fighting forest fires. According to Swiston et al. (2008), CO levels in firefighter work shifts ranged from 5.8–23.2 µg/m^3^, with 6 h levels exceeding 1 mg/m^3^ during the work shift [12]. Adetona et al. (2011) showed that PM_2.5_ concentrations ranged from 5.9 to 2673 µg/m^3^ during prescribed and forest fires [13]. In addition, the actual PM_2.5_ concentrations in our experiment, in the range of 340–1420 µg/m^3^, are comparable to those experienced by women and children in developing countries when using solid fuels for cooking (200 to 3000 µg/m^3^) [21,22] and more than an order of magnitude higher than the concentrations of PM_2.5_ in the atmospheric air of cities under conditions of smoke from forest fires [23,24,25]. 

Most of the air pollution research data from the Indonesian forest fire assessment suggested that particulate matter PM_2.5_ and PM_10_ had been elevated to much more dangerous levels than gaseous compounds [26,27]. In this regard, a significant part of clinical and experimental work was focused on the consequences of the toxic effects of solid particles (PM_2.5_ and PM_10_), while the role of gaseous compounds was considered much less frequently. A few experimental studies have shown that biomass smoke toxicity depends on the type of fuel (oak, peat, pine needles, pine, and eucalyptus) and the combustion phase (flame and smoldering). Kim et al. showed that peat and eucalyptus smoke condensates are more effective in terms of ignition than oak smoke in oropharyngeal aspiration in mice and that material from flaming smoke, with equal mass, is more toxic than from smoldering smoke [28]. Particulate matter is considered to be the best indicator of the health effects of most combustion sources. In female CD-1 mice exposed via oropharyngeal aspiration to PM_2.5_ collected from a real fire at a dose of 100 µg/mouse, exposure to coarse PM collected from a peat fire was shown to cause more severe lung inflammation due to endotoxin and active oxygen species, while ultrafine PM predominantly affected cardiac responses [4]. Martin et al. (2018, 2020) and Thompson et al. (2018) evaluated a single 1 h exposure to peat smoke (PM_2.5_ concentrations of 0.36 mg/m^3^ and 3.30 mg/m^3^, respectively) on the cardiovascular function of white rats. The authors showed that exposure to peat smoke can increase “conditioned susceptibility” to adverse cardiovascular events due to changes in the sensitivity of baroreceptors [29,30,31]. 

The current study showed that continuous exposure to peat smoke for 24 h caused significant changes in behavior and EEG parameters in male rats and also led to a change in these parameters in their F1 offspring. Currently, the mechanisms of the negative effect of smoke components on the human and animal body when inhaled are well-known; however, their toxic effects are often evaluated independently of each other without taking into account the possible combined effect. Neurotoxic effects have been widely studied in acute severe CO poisoning and are characterized by impaired complex brain functions, such as perception, processing and analysis of information, memorization, and learning [32,33,34]. With prolonged exposure to low concentrations of CO, the impairment of the functional state of the nervous system does not correlate with the content of COHb in the blood and can also manifest itself some time after the end of contact [35,36]. At the same time, in the modern literature, there are practically no data on the effect of smoke from natural fires, or its main components, on the state of the nervous system at levels and duration of exposure close to those in real smoke-filled settlements or to occupational exposure.

The present study showed that exposure to peat smoke is associated with increased stress and morphological and neurophysiological changes in the cerebral cortex of rats. Our previous cycle of studies made it possible to reveal some dose–time dependences of the response of rats on exposure to forest fire smoke. Thus, intermittent exposure to smoke in rats for a week for 4 h a day (20 h in total) was accompanied by a significant decrease in locomotor activity and orienting–exploratory behavior against the background of increased anxiety and memory impairment [37]. Prolonged exposure to smoke for 4 weeks, along with a violation of the morphological and functional state of the nervous system, led to a violation of the reproductive function of rats in the form of inhibition of the process of spermatogenesis, changes in the cyclic function of the ovaries, reduced survival, and impaired physiological parameters of their offspring [38]. 

The results of this study showed that the δ-rhythm range prevailed in the encephalogram of the exposed animals compared to the control group, which was more pronounced in the leads of the right hemisphere. Unilateral local slow-wave activity is a sign of a local cortical lesion. An increase in the power of low-frequency rhythms in the δ-band is considered an indicator of the state of anxiety and psycho-emotional stress, which is confirmed by the results of our experiment indicating increased anxiety in animals exposed to smoke. There is a close relationship between EEG power in the β frequency range and metabolic activity in the corresponding cortical region of the brain. It is known that networks of inhibitory interneurons are involved in the generation of β-rhythms [39]. The decrease in spectral power in most high-frequency bands of EEG β-rhythms can be explained by a drop in energy and synaptic activity of the cortex, leading to a weakening of its higher functions [40]. Taking this into account, a decrease in the beta-2 rhythm index may, in our opinion, indicate a change in the activity of inhibitory interneurons, and as a result, cause an increase in the motor and exploratory activities of animals, which was observed after daily exposure to peat smoke.

Histological investigation revealed structural changes in the neurons of the sensorimotor cortex caused by a violation of the supply of oxygen or glucose to cells and characteristic of hypoxic damage to brain tissue [41]. The observed increase in the number of degeneratively altered neurons indicates the general toxic effect of peat smoke, while the appearance of shadow cells is probably a consequence of the hypoxic effect. Given that the blood rheology of the microvasculature of the organ is normal, the hypoxic effect is apparently due to the low oxygen content in the blood of animals. These morphostructural changes in the sensorimotor cortex of exposed rats are probably the bases for behavioral disturbances.

Exposure to peat smoke for 24 h did not affect the spermatogenesis of F0 male rats, as well as the mortality and body weight of F1 offspring in the first month of life. However, pronounced gender behavioral differences were found in adult F1 offspring when tested in an open field. In particular, locomotor hyperactivity in F1 females was evident after paternal exposure to peat smoke. Additionally, there was a change in the bioelectric activity of the brain in F1 male offspring. Previously, we evaluated the effects in the F1 offspring of males exposed to wildfire smoke for 4 weeks. We have shown that reproductive losses (stillbirth and death in the first week of life) when exposed to smoke amounted to 35.7% in females compared to 33% in males. In addition, behavioral and cognitive impairments were found in adult offspring, the severity of which was significantly reduced in offspring obtained by mating 60 days after exposure [42].

It is known that the impact of various environmental and lifestyle factors (diet, physical activity, stress, and smoking) on the father during progenesis can have a significant impact on the development and health of offspring [43,44,45]. Sperm can be epigenetically altered by environmental factors, including PM_2.5_ and wood smoke [8,46]. Clinical studies indicate adverse effects of PM_2.5_ on the male reproductive system [47,48,49,50] and levels of DNA fragmentation in spermatozoa [47,48]. However, the impact of these changes on brain development and offspring behavior remains poorly understood. To date, little is known about the effects of wildfire smoke exposure on the development and health outcomes of future generations, with most studies looking at maternal exposure to smoke in the prenatal period. Two clinical studies [51,52] and one experimental study in monkeys [53] showed that prenatal wildfire smoke exposure resulted in reduced fertility, preterm birth, and reduced birth weight, depending on the time of exposure. Research on the biobehavioral effects of wildfire smoke exposure on offspring is extremely scarce. In the work of Capitanio et al., when observing pregnant female rhesus monkeys and their offspring living outdoors at the California National Primate Research Center during active smoke (California, November, 2018), it was shown that exposure to wildfire smoke (PM_2.5_ concentration was 75.45 µg/m^3^) in the first third of pregnancy has long-term implications for biobehavioral development in baby rhesus monkeys. Specifically, animals exposed prenatally had elevated C-reactive protein levels, decreased plasma cortisol levels, and more passive behavior and memory impairment [54]. Experimental studies by Gorbatova et al. and Ivashova et al. provide data on DNA damage in placental and embryonic cells, as well as impaired behavioral responses in offspring prenatally exposed to rat peat smoke [55,56,57,58]. The authors showed prenatal exposure to peat smoke to lead to a disruption in the formation of a sensorimotor reflex in the early postnatal period, a decrease in the level of natural adaptive fear and intraspecific aggression, and an increase in locomotor activity in mature animals and also considered the possibility of correcting disorders with neuroprotective drugs. 

## 5. Conclusions

Our data indicate that the exposure of males to peat smoke before conception not only leads to impairment of their functional state of the CNS but can also cause sex-specific changes in the behavior and bioelectrical activity of the brain of F1 rats. The results obtained open up a new perspective in the study of neurotoxic and reprotoxic effects associated with the action of wildfire smoke. Considering white rats as a classic object for toxicological research, close to humans in many physiological parameters, we can consider the results obtained as a fundamental step in solving the noted problems through the development of risk-oriented preventive health-saving measures, both for the population and for those working in extreme situations in smoke conditions during natural fires. On the other hand, the revealed relationships between the biological response of the body and exposure to wildfire smoke may contribute to the assessment of the environmental damage of wildfires on populations of small mammals and the potential ability of small mammals to reproduce.

## Figures and Tables

**Table 1:** The content of some combustion products in the air of the exposure chamber.

Pollutant	Mean	Minimum	Maximum	Confidence Interval 95%	Recommended Short-Term (24 h) AQG Level
PM_2.5_,µg/m^3^	920	340	1420	760–1090	15 ^a^
CO, mg/m^3^	40.8	31.5	58.3	38.4–43.3	4 ^a^
NO_2_, µg/m^3^	37	26	49	33–40	25 ^a^
SO_2_, µg/m^3^	2.9	1.0	5.0	2.5–3.2	40 ^a^

^a^—99th percentile (i.e., 3–4 exceedance days per year).

## Data Availability

The data presented in this study are available on request from the corresponding author.
